# Older Adults as Co-Creators in the Research Process: An Overview of Concepts, Methods, and Approaches

**DOI:** 10.1093/geroni/igab046.1504

**Published:** 2021-12-17

**Authors:** Anna Wanka

**Affiliations:** Goethe University Frankfurt, Frankfurt, Hessen, Germany

## Abstract

Participatory approaches have a long-standing tradition in the social sciences and approaches have diversified across multiple research domains. Also in ageing, there is a growing interest in involving older adults, particularly in fields like gerontechnology development, environmental gerontology or patient involvement. In this contribution, ask what participatory approaches and co-creation means in the context of ageing research. What are the benefits and challenges of involving older adults in different research domains, stages of the research process and deploying various participatory methods and approaches? To approach this question, we present preliminary results of a comprehensive scoping review of the literature and provide examples of how older adults can be involved in developing research questions together with researchers, collecting and analysing the data, as well as validating and disseminating study results.

